# The Effects of Low-Dose Remimazolam Adjunct on Propofol–Remifentanil Anaesthesia in Day Case Gynaecological Surgery: A Retrospective Cohort Study

**DOI:** 10.3390/medicina62061177

**Published:** 2026-06-17

**Authors:** Domas Kazokas, Daina Kaveckaitė, Saulė Kraujutaitytė, Ilona Razlevičė, Andrius Macas, Laura Lukošienė

**Affiliations:** 1 Faculty of Medicine, Medical Academy, Lithuanian University of Health Sciences, 44307 Kaunas, Lithuania; 2 Department of Anaesthesiology, Kauno Klinikos, Hospital of Lithuanian University of Health Sciences, 50161 Kaunas, Lithuaniaandrius.macas@lsmu.lt (A.M.); 3 Department of Anaesthesiology, Faculty of Medicine, Medical Academy, Lithuanian University of Health Sciences, 44307 Kaunas, Lithuania

**Keywords:** remimazolam, day case gynaecological surgery, intravenous anaesthesia, recovery, propofol sparing effect

## Abstract

*Background and Objectives:* Recent studies suggest that remimazolam, a novel ultra-short-acting benzodiazepine, has an excellent pharmacokinetic and safety profile, favourable for ambulatory procedures. Although remimazolam has been studied as a sole agent for anaesthesia in day case gynaecological surgery, studies assessing its use in combination with other anaesthetics remain scarce. The aim of this study was to investigate the effects of a low-dose remimazolam adjunct on the characteristics of an intravenous propofol–remifentanil anaesthesia regimen. *Materials and Methods:* A single-centre retrospective observational cohort study was conducted on patients who underwent brief day case gynaecological surgery under general intravenous anaesthesia using remifentanil and propofol from November 2024 to January 2025. The patients were divided into two groups depending on whether they received remimazolam as an adjunct. To account for confounding, propensity scores (PSs) were estimated from baseline characteristics and used to derive stabilised inverse probability of treatment weights (IPTWs). Weighted regression models were then applied to estimate treatment effects on postoperative recovery time measures, consumption of anaesthetics, and incidence of any adverse effects intraoperatively and postoperatively. Cost effectiveness was evaluated using the incremental cost-effectiveness ratio (ICER). *Results:* The clinical data of 51 patients were retrospectively examined: 32 patients were assigned to the intervention group, and 19 patients were assigned to the reference group; after IPTW and PS trimming, the sum of weights was 22 in the intervention group and 58.8 in the reference group. The use of remimazolam as an adjunct was associated with 3.5 min shorter time to eye opening (*p* < 0.001) and 3.6 min shorter time to full consciousness (*p* = 0.002); the total consumption of propofol was decreased by 3 mg/kg (*p* < 0.001); the median dose of remimazolam adjunct was 0.12 mg/kg, or 10 mg per case. There were no statistically significant adverse effects. ICER was 2.35 € per minute of operating room (OR) time saved. *Conclusions:* In the setting of day case gynaecological surgery, the addition of remimazolam to a propofol–remifentanil regimen reduced propofol requirements and shortened recovery time without an increase in adverse effects. This may represent a more efficient anaesthetic approach for ambulatory procedures with a comparable safety profile.

## 1. Introduction

Remimazolam is a novel ultra-short-acting benzodiazepine with a rapid onset and short recovery time. In terms of its sedative effect, remimazolam has been identified as a potential alternative to propofol, additionally possessing a unique advantage of reversibility using the well–established benzodiazepine antagonist flumazenil [1,2]. Recent studies suggest that remimazolam is effective, safe, maintains stable haemodynamics, has a lower rate of respiratory inhibition, reduces pain on injection, and has an excellent quality of postoperative recovery [3,4,5,6]. The aforementioned properties enable a positive integration of this drug into day case surgery, where high patient throughput while maintaining patients’ safety is of key importance. One such instance is brief gynaecological surgeries. Although remimazolam has been studied as a sole agent for anaesthesia induction and maintenance, studies assessing its use in combination with other anaesthetics remain scarce [7,8,9]. Here, therefore, we propose a combinational approach—using remimazolam as an anaesthesia adjunct for anaesthesia induction and maintenance—and describe the implications of such an intravenous anaesthesia regimen in the context of brief day case gynaecological surgeries.

The aim of this study was to investigate the characteristics of an intravenous anaesthesia regimen using remifentanil, propofol, and remimazolam as an adjunct during brief day case gynaecological surgeries, assessing recovery time, doses of anaesthetics needed, and the incidence of adverse effects intraoperatively and postoperatively. We hypothesised that a reasonable combination of remimazolam and propofol could act as a safe, efficient, and cost-effective choice by reducing the relative doses of these agents in comparison to propofol- or remimazolam-only regimens, thus minimising propofol’s cardiovascular depressive effects while maintaining the majority of advantageous properties offered by remimazolam without the need for high consumption of this still high-priced sedative.

## 2. Materials and Methods

### 2.1. Study Design, Inclusion and Exclusion Criteria for Patients, Grouping Method

This was a single-centre retrospective observational cohort study, conducted in accordance with the Declaration of Helsinki, and the protocol was approved by the Regional Biomedical Research Ethics Committee. Adult patients who underwent brief day case gynaecological surgery (hysteroscopy, hysteroscopic abrasion or polypectomy, diathermoconization of the cervix, transobturator tape procedure, endometrial biopsy, and intrauterine device insertion) under general intravenous anaesthesia using remifentanil and propofol from November 2024 to January 2025 were included. Patients with incomplete clinical data were excluded. The patients were divided into two groups depending on whether they received remimazolam as an adjunct: intervention group (received remimazolam as an adjunct) and reference group (no adjuncts were given).

### 2.2. Anaesthesia Regimens and Monitoring

The selection of a particular anaesthesia regimen was determined at the discretion of the anaesthesiologist responsible for its administration.

Intervention group: continuous remifentanil infusion at rate of 0.2 µg/kg/min for the first 10 min followed by continuous infusion at rate of 0.1 µg/kg/min for the remaining time; for induction—IV remimazolam at a dose of 5 mg over 1 min followed by IV propofol titrated at 10 mg every 10 s until loss of consciousness; for maintenance—IV remimazolam bolus doses up to 5 mg as needed, if ineffective, adding a bolus of propofol until effect is achieved.

Reference group: continuous remifentanil infusion at rate of 0.2 µg/kg/min for the first 10 min followed by continuous infusion at rate of 0.1 µg/kg/min for the remaining time; for induction—IV propofol titrated at 10 mg every 10 s until loss of consciousness; for maintenance—IV propofol titrated at 10 mg every 10 s until the desired effect is achieved, with the decision to administer each bolus based on clinical judgement of the patient’s level of consciousness.

All patients received postoperative nausea and vomiting (PONV) prophylaxis with ondansetron according to the institutional protocol.

Monitoring: all the patients had routine non-invasive monitoring of blood pressure, electrocardiogram (ECG), and pulse oximetry (SpO_2_) in the operating room (OR) during the whole duration of anaesthesia; for episodes of respiratory depression, assisted ventilation via face mask with a jaw-thrust manoeuvre was used.

### 2.3. Statistical Analysis

A propensity score (PS) for receiving remimazolam was estimated using a multivariable logistic regression model incorporating the following covariates: age, height, weight, body mass index (BMI), American Society of Anaesthesiologists (ASA) class, and duration of anaesthesia. To ensure adequate overlap between groups, patients with extreme PS were excluded from the analysis; given the relatively small initial cohort, an asymmetric threshold of PS > 0.85 was selected to reduce the influence of extreme weights, while preserving an adequate sample size for analysis. The propensity score model was subsequently re-estimated in the restricted cohort.

Inverse probability of treatment weighting (IPTW) was utilised to reduce the effect of confounding factors between the two groups, estimating the average treatment effect in the treated (ATT). Stabilised weights were used.

The balance of covariates between groups before and after weighting was assessed by standardised mean difference (SMD). An SMD < 0.1 indicated a good balance in the covariates between the two groups.

Time to eye opening in response to patient’s name, time to full consciousness determined as obeying commands, time difference between full consciousness and eye opening, consumption of anaesthetics, incidence of hypotension (MAP < 65 mmHg for at least 1 min), clinically significant bradycardia (HR < 40 bpm, when corrective action is needed), and any adverse effects intraoperatively and postoperatively were evaluated. Time-points were defined as the time from the stop of remifentanil infusion to the time of reaching a certain endpoint. Cost estimation was restricted to medication costs. Personnel costs were not included because staff are compensated via fixed wages independent of time spent in OR at our institution; therefore, differences in OR time between groups were not expected to translate into differences in staffing expenditures at the patient level. Indirect costs, including electricity, maintenance, capital equipment depreciation, and other overhead expenses, were also excluded to simplify the analysis, as these costs are largely fixed over the short time horizon of a single surgical case and their exclusion is unlikely to materially affect comparative results between anaesthesia regimens. The incremental cost-effectiveness ratio (ICER) was estimated as the ratio of the adjusted mean cost difference to the adjusted mean OR time difference between groups, expressed as euros (€) per OR minute saved. Quantitative data were expressed as median [interquartile range]; weighted linear regression was used to estimate adjusted mean differences between groups (treatment effect) with 95% confidence interval (95% CI). Qualitative data were expressed as numbers (percentages); the absolute risk difference between groups with 95% CI was estimated using weighted logistic regression. *p*-values were derived from the Wald test and were regarded as statistically significant when < 0.05. All the analyses were performed using R (version 4.5.2) in RStudio (version 2026.01.1).

## 3. Results

**Figure 1:**
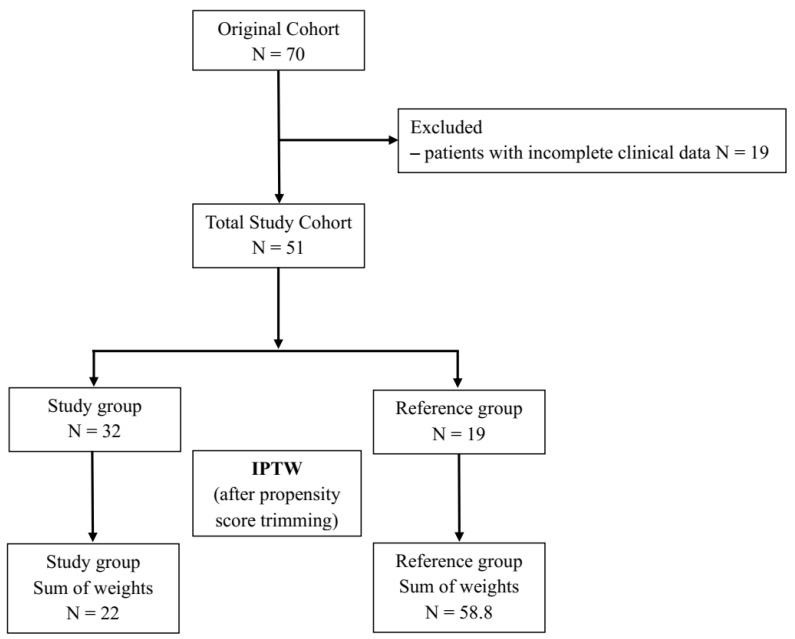
Flowchart for patient identification and exclusion. Abbreviation: IPTW, inverse probability of treatment weighting.

**Figure 2:**
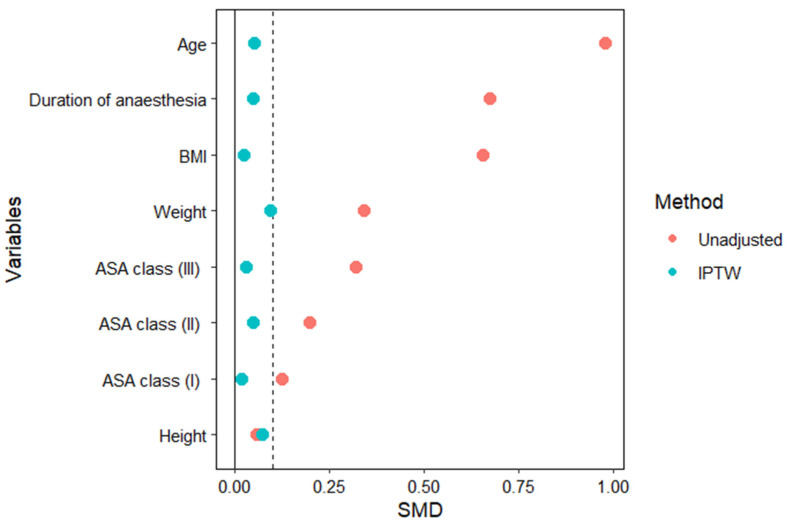
The distribution of standardised mean difference for variables included before and after IPTW adjustment. The dotted line indicates a standardised mean difference of 0.1. Abbreviations: SMD, standardised mean difference; IPTW, inverse probability of treatment weighting; BMI, body mass index; ASA, American Society of Anaesthesiologists.

After the application of the inclusion and exclusion criteria, the clinical data of 51 patients were retrospectively examined. Thirty-two patients were assigned to the intervention group, and 19 patients were assigned to the reference group. After IPTW and PS trimming, the sum of weights was 22 in the intervention group and 58.8 in the reference group (Figure 1). After IPTW adjustment and PS trimming, all covariates were well balanced, with SMDs < 0.1 (Table 1) (Figure 2).

**Figure 3:**
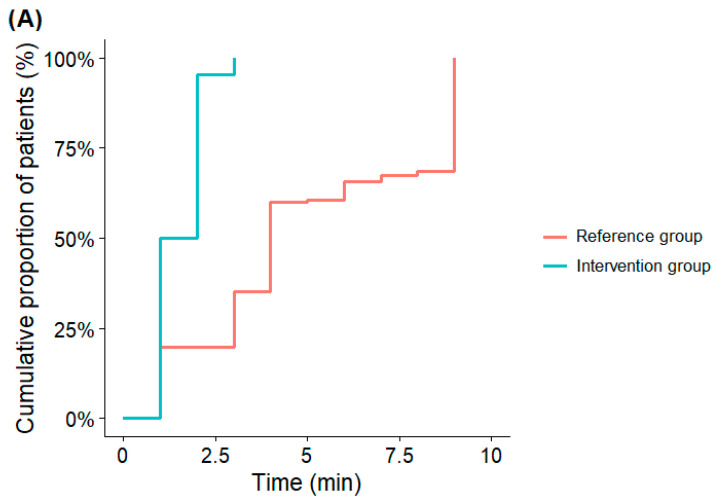

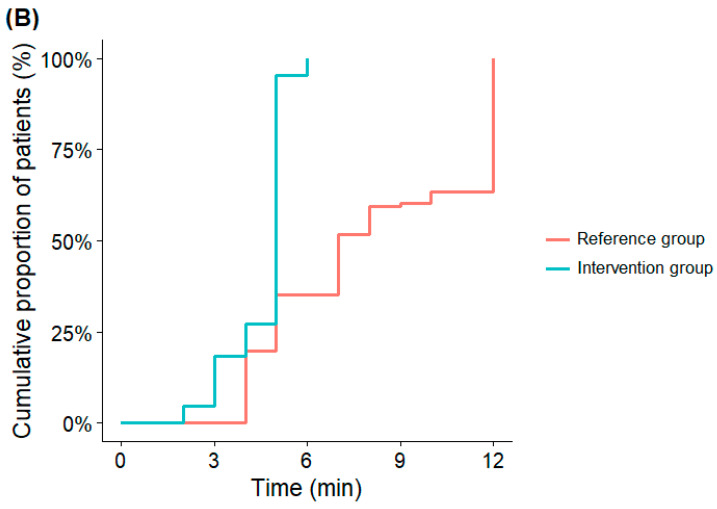
Recovery profiles of different anaesthesia regimens: (**A**) Kaplan–Meier curves of patients who opened eyes at a specific timepoint in response to their name; (**B**) Kaplan–Meier curves of patients who regained full consciousness at a specific timepoint determined as obeying commands.

In IPTW-adjusted analysis, the use of remimazolam as an adjunct was associated with 3.5 min shorter time to eye opening (β = −3.5 min, 95% CI −5.4 to −1.5; *p* < 0.001) and 3.6 min shorter time to full consciousness (β = −3.6 min, 95% CI −5.7 to −1.5; *p* = 0.002); there was no time difference between these two endpoints (β = −0.1 min, 95% CI −0.8 to 0.6; *p* = 0.798). The total consumption of propofol was decreased by 3 mg/kg (β = −3.0 mg/kg, 95% CI −3.4 to −2.5; *p* < 0.001). There was no difference in total remifentanil consumption. The median total remimazolam consumption in the intervention group was 0.12 mg/kg, or 10 mg per case (Table 2). Recovery profiles of different anaesthesia regimens are represented as Kaplan–Meier curves (Figure 3).

The only observed haemodynamic adverse effect was intraoperative bradycardia in the reference group, with an absolute risk of 0.66%, which was not statistically significant (Table 3). Hiccups were observed in three cases of the unweighted, untrimmed study population (n = 32; absolute risk of 9.375%) immediately after the administration of induction agents. All episodes were self-limiting, with a duration of less than 1 min. However, all of these cases were lost during PS trimming and were therefore not included in the final analysis.

With current prices of medication, the estimated ICER, calculated as euros (€) per OR minute saved, was 2.35 €/min (Table 4 and Table 5).

## 4. Discussion

In this single-centre retrospective observational cohort study, we compared two intravenous anaesthesia regimens in day case gynaecological surgeries with respect to recovery profiles, consumption of anaesthetics, and possible adverse effects. We hypothesised that the addition of low-dose remimazolam to the standard propofol–remifentanil regimen would result in reduced doses of propofol required to maintain unconsciousness, thus reducing the incidence of propofol-induced cardiovascular depression and all subsequent adverse effects while maintaining the features of the rapid recovery profile of remimazolam. The basis for this reasoning comes from pharmacokinetic and pharmacodynamic studies: the half-life of elimination for remimazolam is at least twice as short as for propofol, which has higher clearance compared to remimazolam, but a disproportionately larger volume of distribution, mainly due to its high lipophilicity, as well; propofol has dose-dependent effects on the cardiovascular system, even at sedative doses, and results in systemic arterial blood pressure reduction with decrease in cardiac output due to significant decrease in sympathetic tone, leading to reduced vascular resistance and by inhibition of physiological baroreflex responses; in contrast, remimazolam infusion can decrease mean arterial pressure as well, but the effect is less pronounced, reducing mean arterial pressure by up to 20 mmHg while always maintaining systolic blood pressure above 80 mmHg in healthy volunteers [10,11,12]. The reasoning can be justified from the molecular perspective as well: propofol and remimazolam can work synergistically as they act on different binding sites of the same GABA receptor—propofol binds to the β subunit, directly causing the opening of the chloride (Cl^−^) channels, while remimazolam binds to the α subunit, increasing the frequency of Cl^−^ channels opening [13].

Indeed, in our study, we found that adding a low dose of remimazolam significantly decreases total propofol consumption. With remimazolam used as an adjunct, we observed shorter times to both eye opening and full consciousness. However, there was no significant difference in the interval between these two endpoints, suggesting that using the anaesthesia regimen we have described, remimazolam primarily influences the initiation of emergence (awakening), whereas the subsequent progression of recovery is not altered. This interpretation is supported by the recovery profiles presented as Kaplan–Meier curves, in which the cumulative proportion of patients reaching predefined endpoints was used as a population-level surrogate of level of consciousness over time: in the curve depicting time to eye opening, the intervention group demonstrated a steeper slope, indicating faster initial emergence, whereas in the curve depicting time to full consciousness, the distribution was primarily shifted leftward along the time axis. Taken together, this pattern implies that the addition of remimazolam shifts the recovery trajectory earlier in time without modifying its intrinsic dynamics—patients awaken sooner, but once awakening has occurred, the rate of recovery to full consciousness proceeds similarly regardless of whether remimazolam was used.

Studies combining propofol and remimazolam for day case hysteroscopy consistently showed lower propofol consumption [13,14,15]. The minimum propofol-sparing dose of remimazolam was 0.1 mg/kg, and the mean 50% effective dose of remimazolam was 0.09 (95% CI 0.08–0.11) mg/kg, which is consistent with the total remimazolam consumption empirically found in our study [16,17]. The data on recovery times is heterogeneous, which could be attributed at least partly to different anaesthesia regimens used in the studies. For instance, in a study where propofol was used as an adjunct and remimazolam was administered as a continuous infusion, the postoperative recovery time was significantly shorter than in the propofol-only regimen [13]. In other studies, where remimazolam was used as an adjunct, there was no difference in the postoperative recovery time between groups; however, in all these studies, propofol was administered as a continuous infusion rather than boluses, which resulted in a higher total propofol consumption compared to our regimen [14,16,17]. Interestingly, studies combining propofol and remimazolam in gastroscopy, where the medications were administered as boluses (a regimen similar to ours), found that the combination group had the shortest recovery time compared to the propofol-only or remimazolam-only groups, or at least it was the same as in the remimazolam-only group [18,19].

Studies have shown that a combination of remimazolam and propofol is associated with less intraoperative hypotension than propofol alone, although the difference was not always statistically significant; it is worth mentioning, that the definition of intraoperative hypotension varied widely among these studies as well as the incidence of hypotension itself, ranging between 2.5 and 15.4% in combination groups compared to 15.0–42.2% in propofol-only groups [13,14,15,16,17]. The absence of hypotension in either group in our study could be attributed to the small sample size and the relatively liberal definition of intraoperative hypotension. The findings of our study regarding intraoperative bradycardia are consistent with those of other studies. In some studies, bradycardia was more prevalent in propofol–only groups than in combination groups, but no statistically significant differences were observed [13,14,16]. Although gynaecological procedures are associated with one of the highest risks of PONV among surgical specialties, we did not observe any cases of PONV [20]. This could be due to the relatively small size of our cohort, which may have impacted the ability to detect low-frequency adverse effects, particularly given the use of prophylaxis. Whether remimazolam reduces the incidence of PONV remains controversial. While it seems that the use of remimazolam is associated with a lower incidence of PONV compared to inhalational anaesthesia, it is inferior to propofol in preventing postoperative vomiting [21]. However, it might have antiemetic properties as flumazenil antagonism of remimazolam increases the incidence of PONV, and the addition of low-dose remimazolam is associated with a lower incidence of nausea at 6 h postoperatively [22,23]. The mechanism by which remimazolam induces hiccups remains unclear. The incidence of hiccups in our study was consistent with that reported by other authors, in the range of 5.6–10.0% [14,15]. Hiccups caused by remimazolam are more likely to occur with rapid administration of remimazolam compared to slower infusion, and one recent study found that lower doses of remimazolam might be associated with a higher incidence of hiccups compared to higher doses [24,25]. Nevertheless, they are usually self-limiting [26]. However, if they were to persist, a number of methods can be used to terminate it. Firstly, remimazolam should be discontinued and an alternative agent for maintaining unconsciousness and deepening anaesthesia, such as propofol, should be used instead [27]. Secondly, non-pharmacological measures should be implemented: nasopharyngeal stimulation at the C2 and C3 vertebrae through the insertion of a suction catheter or the application of a local anaesthetic-lubricated nasopharyngeal airway, a continuous positive airway pressure (CPAP) between 25 and 35 cmH_2_O for 5 to 15 s, an increase in the pressure of carbon dioxide in the alveoli [28,29,30,31]. Thirdly, pharmacological agents could be used: as remimazolam’s effects can be reversed with flumazenil, the latter might be used, as it has been successfully administered to terminate midazolam-induced hiccups [32]. Other medications for the management of hiccups include chlorpromazine, metoclopramide, ephedrine, dexmedetomidine, and lidocaine [31]. Lastly, muscle relaxants could be used in cases of intractable hiccups, but it requires proper airway management measures [27].

This study did not include a control group using only remimazolam, as there were no cases of this regimen at our institution. However, another study found that the total consumption of remimazolam in such a regimen was 0.36 ± 0.07 mg/kg for an approximately 12 min procedure [13]. In comparison, the total consumption of remimazolam in our combinational regimen was only 0.12 (95% CI 0.10–0.15) mg/kg for an approximately 35 min procedure. Currently, there is no robust cost-effectiveness analysis of combinational anaesthesia regimens using remimazolam in the literature, but it is very likely that a combinational regimen could be cost-effective, as it provides shorter recovery, maintains a better safety profile compared to a propofol-only regimen (as shown in the literature), with lower total consumption of remimazolam compared to remimazolam-only anaesthesia. In many cases, the ICER value we found would be lower than the cost per OR minute, but the precise impact depends on the local financing policy of procedures and institutional financial management. However, the regimen we used definitely has the potential to increase patient turnover, which would generate a larger overall income for the healthcare institution. For instance, in our hospital, with an OR working 7 h a day, 10 min for patient positioning, peripheral vein catheterization and monitoring initiation, a standard anaesthesia duration of 35 min, a mean full recovery time of 8.1 min (the value we found in the reference group) and 20 min for OR preparation for the next case, a maximum of 5 (5.75) such procedures can be performed per day. Using remimazolam results in a 3.6 min shorter recovery time for each case, which would ultimately allow an additional procedure to be performed. The estimated expenditure for this additional time using remimazolam would be ~60 € per day, while an additional day-case gynaecological procedure generates ~500 € of income for an institution.

Future research could include optimal anaesthesia regimens combining propofol and remimazolam for day case gynaecological surgery and regimens for specific subpopulations, such as obese patients and the elderly, implications of different methods of administering medication (continuous infusion vs. boluses), comparison of recovery profiles using anaesthesia depth monitoring, solid cost-effectiveness analysis of using combination regimen, studies evaluating long-term anaesthesia-related outcomes and fundamental research of some adverse effects of remimazolam, such as hiccups, which can be clinically significant in patients at increased risk of regurgitation and aspiration.

This study had several limitations. Firstly, a control group using the remimazolam-only regimen is necessary for a comprehensive comparison between regimens. Secondly, the sample size was relatively small. Thirdly, as an observational study, there is a risk of unmeasured confounding not included in the multivariable logistic regression model used to calculate PS. It may include, but are not limited to, factors that could influenced treatment allocation (individual anaesthesiologist preference for anaesthesia regimen, experience and familiarity with remimazolam, perceived patient frailty not fully reflected by ASA status or recorded comorbidities), subtle differences in patient clinical status not captured by medical records, the complexity of a surgical procedure, temporal factors (for instance, increasing clinicians’ experience with remimazolam over time at our institution). Therefore, findings of this single-centre retrospective observational cohort study, susceptible to all the limitations of this study design, require validation through multicentre randomised controlled trials.

## 5. Conclusions

In the setting of day case gynaecological surgery, the addition of remimazolam to a propofol–remifentanil regimen reduced propofol requirements and shortened recovery time without an increase in adverse effects. This regimen may represent a more efficient anaesthetic approach for ambulatory procedures with a comparable safety profile.

## Figures and Tables

**Table 1:** Patient characteristics for before IPTW adjustment and after IPTW adjustment.

	Before IPTW Adjustment			After IPTW Adjustment		
Variables	Intervention Group n = 22 (56.4%)	Reference Group n = 17 (43.6%)	SMD	Intervention Group	Reference Group	SMD
**Age (years)**	50.0 [44.2–56.0]	56 [50–69]	0.9794	50.0 [44.2–56.0]	50 [43–50]	0.0492
**Height (cm)**	168.0 [164.0–169.8]	168 [165–168]	0.0555	168.0 [164.0–169.8]	168 [167–168]	0.0727
**Weight (kg)**	68.8 [61.5–88.0]	73 [66–90]	0.3424	68.8 [61.5–88.0]	70 [63–73]	0.0927
**BMI (kg/m^2^)**	24.5 [22.8–30.2]	28.2 [25.9–33.1]	0.6550	24.5 [22.8–30.2]	26.2 [22.0–26.9]	0.0242
**ASA class**						
**I, n (%)**	4 (18.2%)	1 (5.9%)	0.1230	18.2%	16.5%	0.0172
**II, n (%)**	16 (72.7%)	9 (52.9%)	0.1979	72.7%	77.4%	0.0472
**III, n (%)**	2 (9.1%)	7 (41.2%)	0.3209	9.1%	6.1%	0.0300
**Duration of anaesthesia (min)**	32.5 [30.0–40.0]	45 [35–45]	0.6753	32.5 [30.0–40.0]	35 [30–45]	0.0474

Abbreviations: IPTW, inverse probability of treatment weighting; SMD, standardised mean difference; BMI, body mass index; ASA, American Society of Anaesthesiologists.

**Table 2:** Comparison of outcomes between different anaesthesia regimens.

Outcome		Intervention Group	Reference Group	β (95% CI)	*p*-Value
**Time to eye opening (min)**		1.5 [1.0–2.0]	4.0 [3.0–9.0]	−3.5 (−5.4 to −1.5)	<0.001
**Time to full consciousness (min)**		5.0 [4.2–5.0]	7 [5–12]	–3.6 (−5.7 to −1.5)	0.002
**Time difference between eye opening and full consciousness (min)**		3 [2–4]	3 [3–3]	−0.1 (−0.8 to 0.6)	0.798
**Total propofol consumption**	**(mg)**	75.0 [52.0–100.0]	300 [250–300]	–209.4 (−254.6 to −164.2)	<0.001
	**(mg/kg)**	1.1 [0.9–1.5]	4.1 [4.0–4.7]	–3.0 (−3.4 to −2.5)	<0.001
**Total remifentanil consumption**	**(µg)**	245.0 [136.2–268.8]	196.0 [84.6–200.0]	52.8 (−7.4 to 112.9)	0.084
	**(µg/kg)**	2.7 [2.4–3.9]	3.0 [1.0–3.2]	0.6 (−0.5 to 1.7)	0.246
**Total remimazolam consumption**	**(mg)**	10 [7–10]	–	–	–
	**(mg/kg)**	0.12 [0.10–0.15]	–	–	–

Abbreviations: β, adjusted mean difference estimated using a weighted linear regression model; CI, confidence interval.

**Table 3:** Incidence of adverse effects.

Outcome	Intervention Group	Reference Group	Risk Difference (95% CI)	*p*-Value
**Intraoperative hypotension**	0%	0%	–	–
**Intraoperative bradycardia**	0.00%	0.66%	−0.66% (−2.13% to 0.81%)	0.367
**Postoperative hypotension**	0%	0%	–	–
**Postoperative bradycardia**	0%	0%	–	–
**Postoperative nausea**	0%	0%	–	–
**Postoperative vomiting**	0%	0%	–	–

Abbreviations: CI, confidence interval.

**Table 4:** Current prices of medication.

Medication	Price (€)	Quantity	Price per Quantity
**Propofol**	1.00	Vial of 200 mg	0.005 €/mg
**Remifentanil**	7.00	Vial of 1 mg	0.007 €/µg
**Remimazolam**	20.00	Vial of 20 mg	1.000 €/mg

**Table 5:** Data used for ICER estimation.

Outcome	Weighted Mean in Intervention Group	Weighted Mean in Reference Group	Adjusted Difference (95% CI)
**Cost (€)**	11.03	2.57	8.46 (7.10 to 9.81)
**Time to full consciousness (min) ^1^**	4.5	8.1	−3.6 (−6.7 to −0.43)

Abbreviations: ICER, incremental cost-effectiveness ratio; OR, operating room; CI, confidence interval. ^1^ Time to full consciousness served as a proxy for OR utilisation time.

## Data Availability

The data supporting the findings of this study are available from the corresponding author upon reasonable request.

## Abbreviations

The following abbreviations are used in this manuscript:

ASA	American Society of Anaesthesiologists
BMI	Body mass index
BPM	Beats per minute
CI	Confidence interval
CPAP	Continuous positive airway pressure
GABA	γ–aminobutyric acid
HR	Heart rate
ICER	Incremental cost-effectiveness ratio
IPTW	Inverse probability of treatment weighting
IV	Intravenous
MAP	Mean arterial pressure
OR	Operating room
PS	Propensity score
PONV	Postoperative nausea and vomiting
